# Clinicopathological characteristics and outcomes of metachronous rectal cancer in patients with a history of cervical cancer with and without remote radiotherapy: Reports of 45 cases

**DOI:** 10.1097/MD.0000000000021328

**Published:** 2020-07-24

**Authors:** Hsinyuan Hung, Jengfu You, Jyming Chiang, Paoshiu Hsieh, Sumfu Chiang, Chengchou Lai, Wensy Tasi, Chienyuh Yeh, Yihjong Chern, Yujen Hsu

**Affiliations:** Division of Colon and Rectal Surgery, New Taipei Municipal TuCheng Hospital, Chang Gung Memorial Hospital, Chang Gung University College of Medicine, 5, Fu-Hsing St., Kuei-Shan, Tao-Yuan, Linko, Taiwan.

**Keywords:** cervical cancer, prognosis, radiotherapy, rectal cancer

## Abstract

The purpose of this study was to report the clinicopathological characteristics and treatment outcomes of 45 rectal cancer patients who have a history of cervical cancer with or without remote radiotherapy. Twenty-nine patients (64.4%) with a history of cervical cancer treated with pelvic radiotherapy were classified as group A, 16 (35.6%) patients with a history of cervical cancer not treated with radiotherapy were classified as group B. The median duration between radiotherapy for cervical cancer and rectal adenocarcinoma diagnosis was 18 years. At the time of rectal cancer diagnosis, 5 (17.2%) patients presented stage I disease, 15 (51.7%) had stage II, 1 (3.4%) had stage III, and 8 (27.6%) had stage IV. The patients in group A had older age, higher rates of gross ulcerative lesions, low hemoglobin levels, and a lower rate of lymph node metastases. The patients with secondary rectal cancer developed after radiotherapy for cervical cancer usually presented with abnormal abdominal symptoms, such as proctitis, cystitis, or rectal fistula. Higher colostomy rate was found in this group of patients due to severe pelvic fibrosis or proctitis.

## 1 Introduction

The prolonged cancer survival that has been achieved through advances in multidisciplinary cancer treatments. The incidences of secondary malignancies are also increasing. Secondary cancers typically occur a very long time after radiation therapy, and the interval between radiotherapy and occurrence of a second primary malignancy is often greater than 10 years.^[1,2]^ Thus, the influences of radiotherapy, as a cancer treatment, on the risk of secondary malignancies had been investigated.^[3]^

In the literature, the first observed case of rectal cancer that developed after pelvic radiotherapy was described by Slaughter and Southwich in 1957,^[4]^ after which Castrio reported another 26 cases of colorectal cancer that developed after radiotherapy for cervical and uterine cancers in 1972.^[5]^ Following these observations, other secondary malignancies that have been induced by radiotherapy, including leukemia, sarcomas, thyroid carcinomas, and lung carcinomas, have also been reported.^[6,7]^

In recent years, several large epidemiologic studies years have also identified the significantly increased incidence of colorectal cancer after pelvic radiotherapy for cervical cancer, and the risk of radiation-induced tumor formation continues to the end of life.^[2,8–10]^ Boice et al reported 162 of 3,324 (5%) second cancers following radiation treatment for cervical cancer could be attributed to radiation.^[2]^ Kleinerman et al found an increased risk (relative risk = 1.4) of secondary malignancy near site of radiation in 5997 cervical cancer patients who had received radiotherapy.^[8]^ Teng et al found the radiotherapy for cervical cancer had a deferent impact on secondary malignancy risk (1.41 hazard ratio).^[10]^ A large number of female patients with cervical cancer have received prior radiotherapy, and many of whom have also achieved a long period of survival. A long time after radiotherapy, some patients present secondary malignancies during follow-up, and these patients provide an excellent opportunity for studying incidences and clinicalpathologic characteristics of radiation-related secondary malignancies. However, to the best of our knowledge, only a few case reports have discussed these clinical features and treatment outcomes. Similarly, several large epidemiologic studies using a population-based cohort only presented relative risk and type of secondary malignancies. No studies have focused on secondary rectal cancer and compared the detailed differences in clinicopathological factors between patients with rectal cancer with or without previous radiotherapy for cervical cancer. Therefore, we retrospectively investigated the clinicopathological characteristics and treatment outcomes of female patients with secondary rectal cancer who previously received radiotherapy for cervical cancer, compared with those who did not receive radiotherapy for cervical cancer.

## 2 Materials and methods

### 2.1 Patients

This study was approved by the Institutional Review Board (IRB) of Chang Gung Memorial Hospital in Taiwan. For this retrospectively study, patient data from January 2000 to December 2015 was obtained from the colorectal cancer registry database of a single medical center hospital. A total of 45 female patients with primary cervical cancer and secondary rectal adenocarcinomas were enrolled in this study. We defined 2 groups for analysis: (1) group A: 29 patients (64.4%) with a history of cervical cancer treated with radiotherapy, (2) group B: 16 patients (35.6%) with a history of cervical cancer treated without radiotherapy. The tumor/node/metastasis (TNM) classification and staging was performed in accordance with the 7th American Joint Committee on Cancer (AJCC) cancer staging guidelines.^[11]^

### 2.2 Patients

We compared clinicopathological characters and treatment outcomes between the 2 patient groups. The following variables were analyzed: (1) patient factors, including age, carcinoembryonic antigen (CEA) levels, hemoglobin levels, albumin levels, and treatment and survival outcomes and (2) tumor and pathologic factors, including tumor size, gross appearance, histologic subtype, histologic grade, and TNM stage.

### 2.3 Follow up

All patients received regular follow-up after the rectal cancer operation. Each follow-up visit included a physical examination and CEA test. Abdominal ultrasonography, computer-assisted tomography from the chest to pelvis, and colonoscopy exams were arranged every 1 to 3 years, depending on each patient's clinical condition.

### 2.4 Statistical analysis

Overall survival (OS: the proportion of cancer patients who survived for a specified time interval after surgery) data was calculated using the Kaplan-Meier method. OS was calculated since the diagnosis of rectal cancer. The effect of each prognostic factor was examined using the log-rank test. Cox proportional hazard models and regression analyses were performed using the Statistical Package for the Social Sciences, release 17.0 (SPSS Inc. Chicago, IL). Comparisons between groups were made using the χ^2^ test. A *P* value less than .05 was considered statistically significant.

## 3 Results

### 3.1 Patient characteristics

The pertinent clinical details of all 29 patients who developed rectal cancer after radiotherapy (group A) for cervical cancer are listed in Table 1. The median duration between radiotherapy for cervical cancer and rectal adenocarcinoma diagnosis was 18 years, with a range of 3 to 44 years. At the time of rectal cancer diagnosis, 5 (17.2%) patients presented stage I disease, 15 (51.7%) had stage II, 1 (3.4%) had stage III, and 8 (27.6%) had stage IV. We suspected rectal cancer owing to symptoms of the presence of a bloody or mucoid stool (13 patients, 44.8%), abdominal pain/diarrhea (8 patients, 27.6%), CEA elevations during cervical cancer follow-up (7 patients, 24.1%), and the presence of rectovaginal fistula (2 patients, 6.9%). Only 1 patient had bone metastases from the cervical cancer at the time she was diagnosed with a rectal cancer; the other 28 patients had no recurrence of the cervical cancer. The radiation regimen and dose used for the cervical cancer treatment were not available in some cases because of the long interval and unavailable previous records. The rate of radiotherapy for the rectal cancer was relatively low due to the risk of cumulative radiation toxicity; only 3 (10.3%) patients received neoadjuvant radiotherapy (25, 25, and 50.4 Gy, respectively). Nine (33.3%) patients received adjuvant or palliative chemotherapy.

**Table 1:**
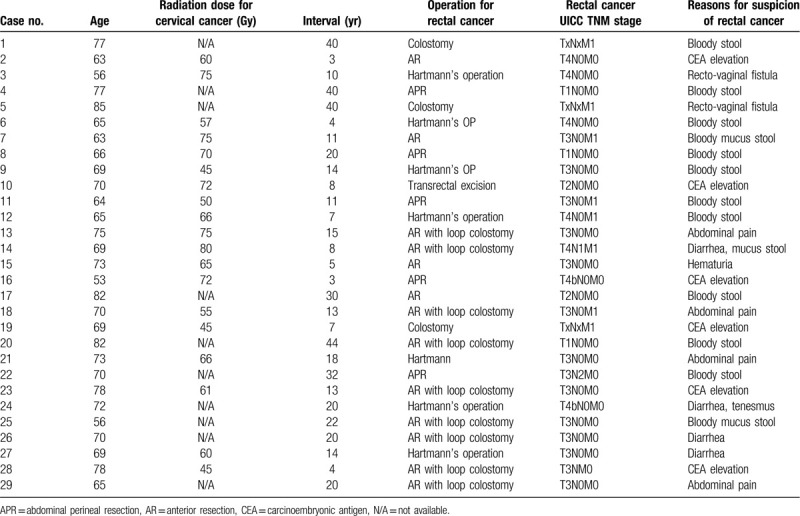
Summary of 29 cases who developed rectal adenocarcinomas after radiotherapy for cervical cancer (group A).

The clinical and pathological characteristics of the 45 female patients in the 2 groups are shown in Table 2. The patients in group A were significantly older than group B. The median patient age was 70.0 years (range, 53-85 years) for group A and 64.0 years (range, 37–79 years) for group B. The median follow-up duration after rectal cancer surgery was 49 months, with a range of 12 to 146 months. Regarding the clinical characteristics, the patients in group A had higher rates of gross ulcerative lesions (88.0% vs 62.5%, *P* = .040), hemoglobin levels <10 g/dL (31.0% vs 0%, *P* = .011), and a lower rate of lymph node metastases (8% vs 43.7%, *P* = .008).

**Table 2:**
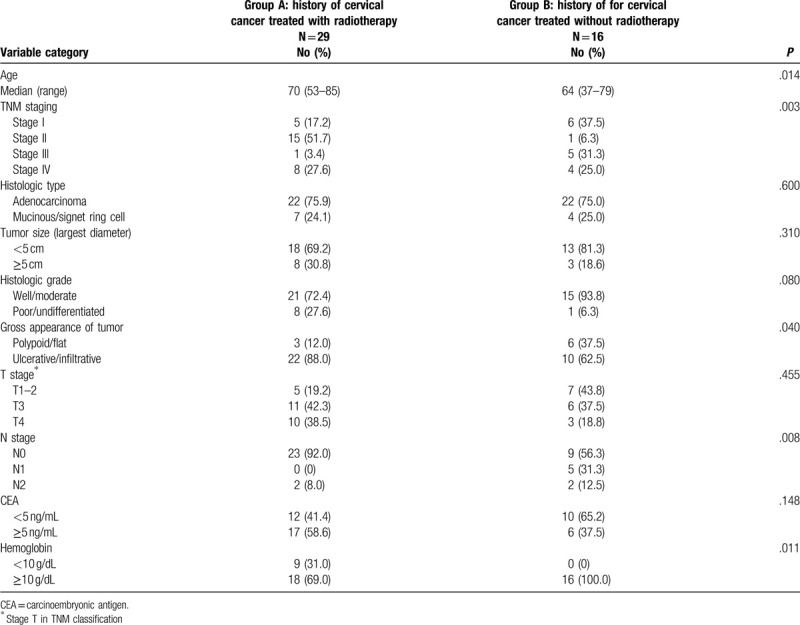
Clinical and pathological characteristics of 45 female patients with rectal cancer.

### 3.2 Clinical presentations

**Figure 1:**
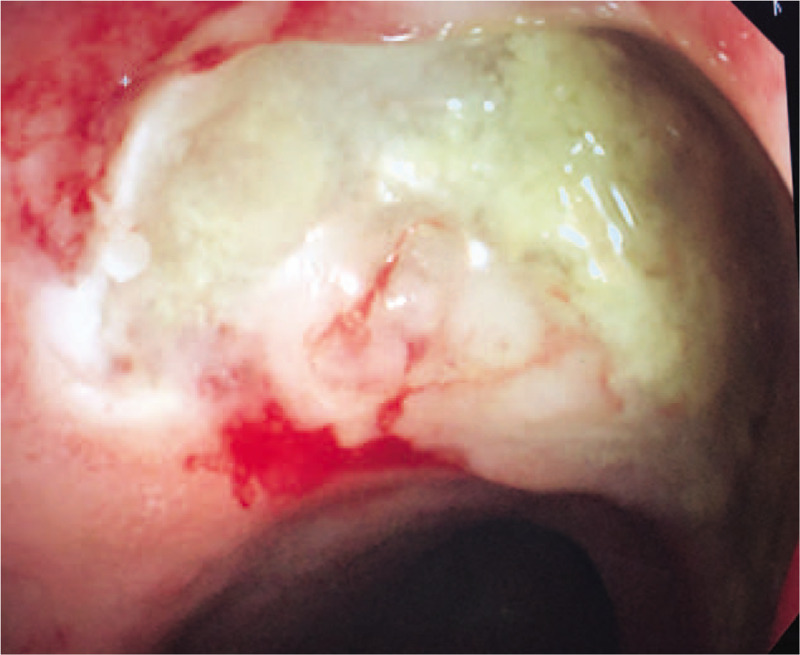
Secondary rectal adenocarcinoma presented with large deep ulceration but no grossly tumor.

**Figure 2:**
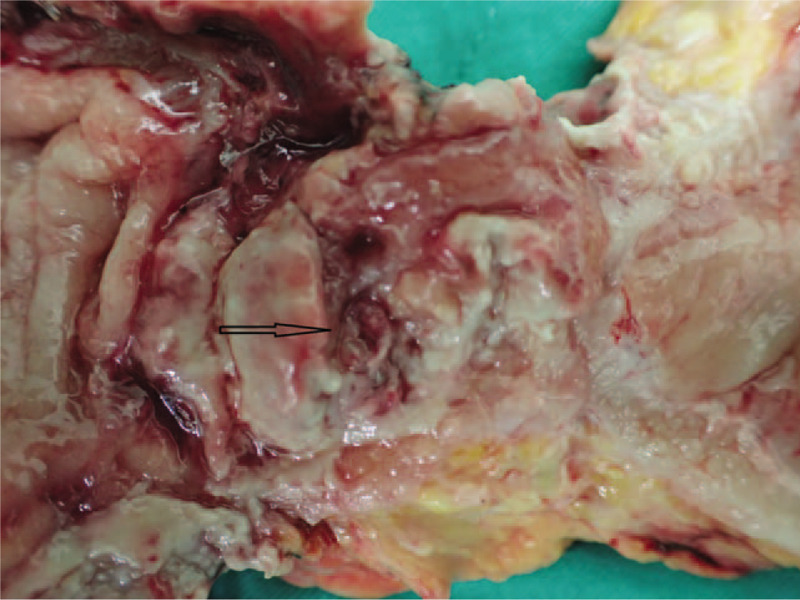
Secondary rectal adenocarcinoma presented with fistula between rectum and urinary bladder (arrow), no typical gross ulcerated tumor.

The patients in group A displayed a variety of clinical presentations. Three patients did not display gross tumor-like lesions. In one of the patients, only a deep ulceration at the middle rectum was found during colonoscopy (Fig. 1). Fistula formations between the rectal tumors and adjacent organs were found in 2 of the patients; 1 rectovaginal and 1 rectovesical fistula. Although the histopathologic analysis revealed a rectal adenocarcinoma, no typical gross ulcerated tumor was identified at the resected rectum in the patient with a rectovesical fistula (Fig. 2). Since most cases had severe pelvic chronic inflammation (severe fibrosis 27.6%, proctitis or cystitis 41.4%), poor healing of rectal anastomoses and high anastomosis leakage rates were expected, and a high colostomy rate was needed in this group. In group A, 21 patients (72.4%) required colostomies; 11 (37.9%) patients had permanent and 10 (34.5%) had temporary colostomies. The permanent stoma rate in this group was higher than that in group B (25%).

### 3.3 Survival of patients

**Figure 3:**
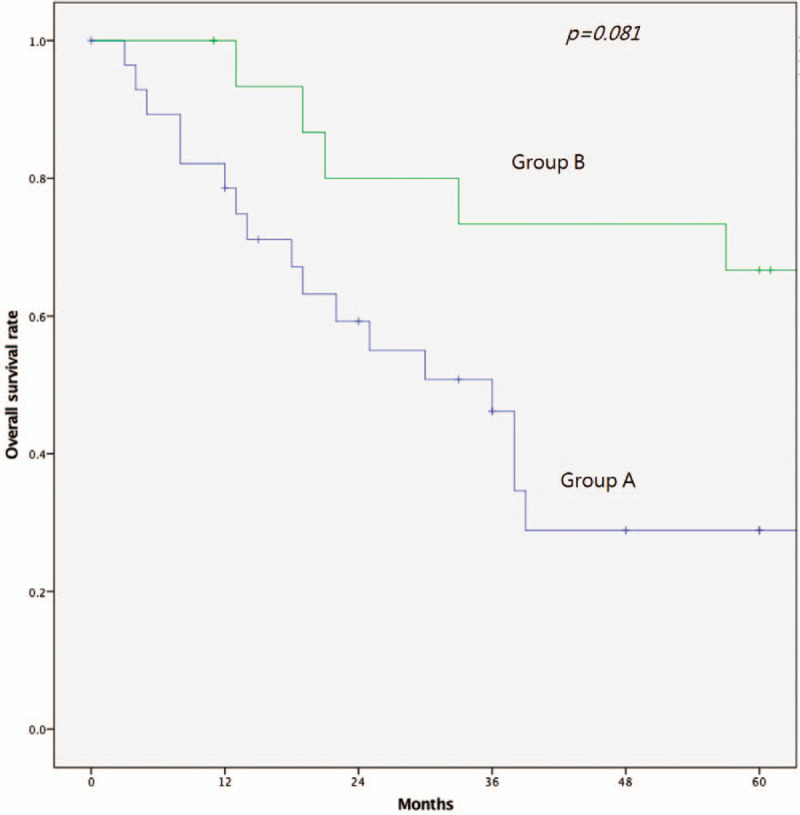
Kaplan-Meier survival analysis. Overall survival of 2 groups.

The OS curves are presented in Fig. 3. The patients in group A had worse OS than those in group B but not reach statistical significance (5-year OS rate: 28.7% vs 67.2%, *P* = .081, Fig. 3). The adjuvant treatment and final follow-up statuses of the 3 groups are listed in Table 3. In group A, only 34.5% of the patients remained disease-free, and 8 (27.6%) patients presented with a stage IV cancer at the time of diagnosis; 5 had liver metastases, 1 had a lung metastasis, and 2 had pelvic organ metastases. Ten (34.5%) patients developed recurrence during the follow-up; 7 patients had pelvic recurrence, 1 had intra-abdominal recurrence, and 2 had inguinal lymph node metastases. None of the 7 patients with pelvic recurrence received adjuvant radiotherapy for the secondary rectal cancer; however, 3 of them received adjuvant chemotherapy. The results of the multivariate analyses of the factors affecting the OS were shown in Table 4. Cox regression analysis revealed that age ≥ 65 years, poorly differentiated tumor classification and TNM stage 4 and patients with remote were independent predictors of both OS. Patients with previous radiotherapy for cervical cancer was not significant predictors for OS in multivariate analyses.

**Table 3:**
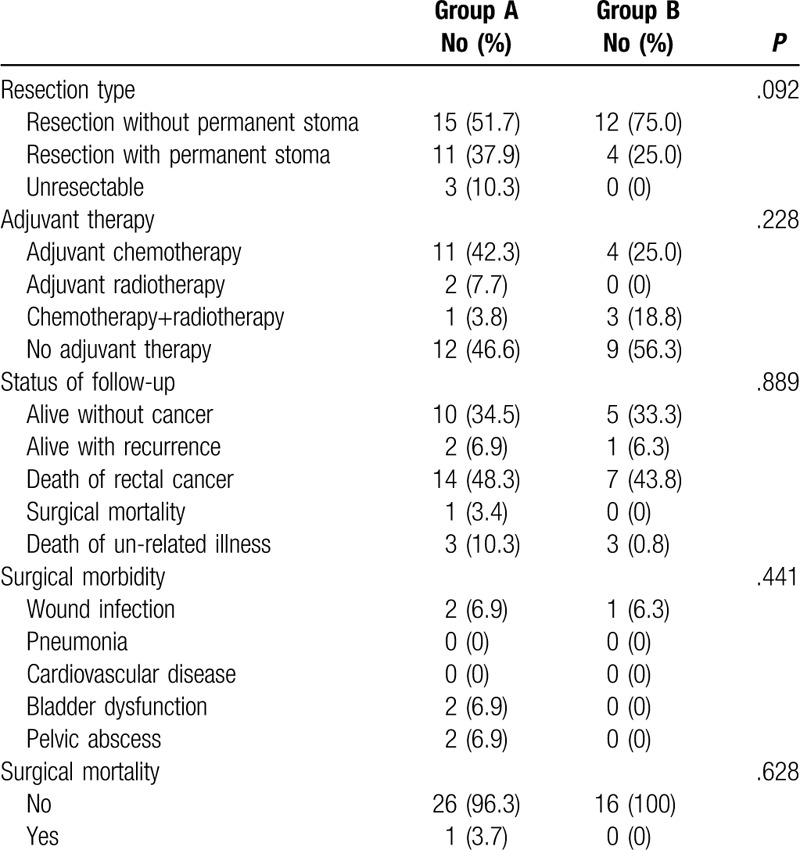
Resection type and outcomes status.

**Table 4:**
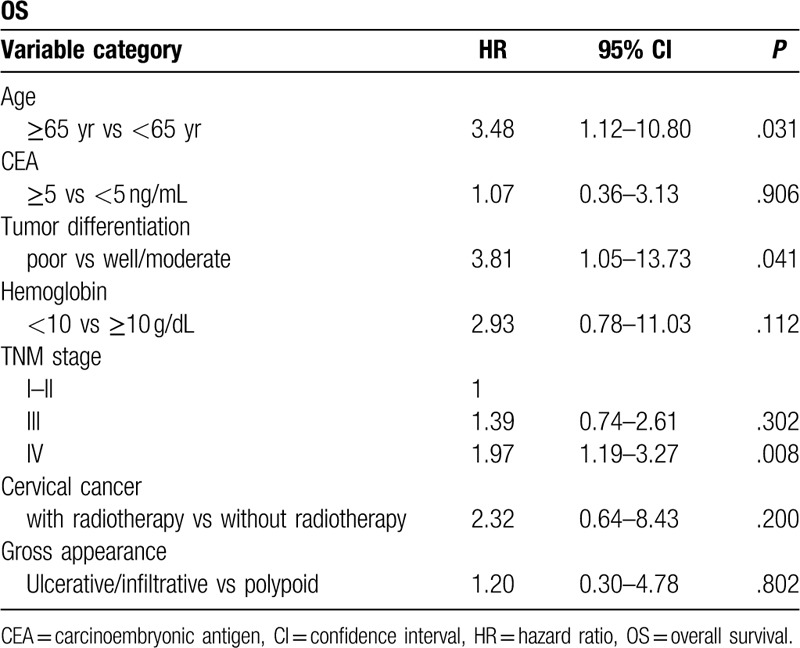
Multivariate analysis of prognostic factors for overall survival of 45 females with rectal cancer.

## 4 Discussion

Radiotherapy is a potential carcinogenic treatment, and the purpose of pelvic radiotherapy is to reduce cancer recurrence in the pelvic region. However, the radiation injury caused by pelvic radiotherapy also increases the formation of secondary cancers, which are observed after long follow-up durations. The increased incidence of rectal cancer in patients with cervical cancer has been reported in large-scale observational studies.^[12]^ In Taiwan, a cohort study that used nationwide population data enrolled 35,175 patients with cervical cancer and found that 268 (0.8%) patients developed secondary rectal or colon cancer. The hazard ratio of secondary malignancies risk post pelvic radiotherapy was 1.27 (1.14–1.43).^[10]^

In the present study, we identified 29 female patents with rectal cancer who previously received pelvic radiotherapy for cervical cancer. The mean duration from radiotherapy to rectal cancer detection was 18 years, and this observation is comparable with those of other studies. According to the literature, the duration between radiotherapy and development of secondary cancer within the radiated area is usually greater than 10 years.^[13,14]^

However, whether a direct relationship exists between pelvic radiotherapy and the formation of secondary rectal cancer remains controversial, and opposing opinions have been presented in previous studies. Neugut et al found an association of radiotherapy of the prostate with bladder cancer (1.5-fold risk), but not with rectal cancer (0.8-fold risk), upon analyzing the Surveillance, Epidemiology, and End Results (SEER) data (1973–1990) published in 1997.^[6]^ Another study analyzed 201,438 patients with prostate cancer who received or did not receive pelvic radiotherapy and found that increased rectal cancer risk was associated with age and risk factors other than pelvic radiotherapy.^[15]^

In contrast, several later studies derived different conclusions from analyses of large-scale epidemic datasets. Baxter et al used SEER data (1973–1993) that was published in 2005 and found a significant increase in rectal cancer development post prostate radiotherapy, with a 1.7 hazard ratio compared with the surgery-only group, and the risk increases with survival.^[16]^ Another large-scale study of Ruth et al also supported this observation and reported a 1.7-fold risk for secondary rectal cancer across 86,193 patients with cervical cancer.^[8]^ The secondary cancer interval is usually longer than 10 years, and we hypothesize that inadequate follow-up periods could lead to underestimated secondary rectal cancer risks.

Although cervical cancer can be cured by radiotherapy, the associated radiation causes injuries including small vessel occlusions, tissue fibroses, repeated inflammation at the radiated area, and rectovaginal fistula formations. Many pelvic radiation-induced complications have been reported, such as intestinal obstructions, perforations and fistulas, arterial or venous occlusions, and pelvic bone fractures.^[17–19]^ The reasons for these complications are related to chronic ischemia and the fibrosis of pelvic organs after pelvic radiotherapy, and these side effects predominantly localize to the rapidly reproducing tissue located in the bowel and bladder mucosa. The most common clinical presentations after pelvic radiotherapy for cervical cancer are proctitis or cystitis, and patients were also observed to present with urine or stool urgency and blood found in the stool or urine. Eifel PJ reported that the overall risk of major complications for patients who received pelvic radiotherapy for cervical cancer has been as high as 12.9% at 10 years after radiotherapy, and the overall risk of fistula between pelvic organs was 3.1%.^[17]^ Our results were compatible with previous reports, and in our series, rectovaginal fistulas were found in 6.9% of patients, severe pelvic fibrosis in 27.6%, and proctitis and/or cystitis in 41.4%.

The mechanism of carcinogenesis post radiotherapy remains controversial.^[20]^ Dasu and colleagues analyzed risk estimation models and suggested that dose inhomogeneity is associated with secondary cancer formation. Tsuji et al analyzed genetic changes in radiation-associated rectal cancer and found that carcinogenesis occurs through a multistep tumorigenesis pathway, and loss of heterozygosity and genomic instability are related to malignancy development. These authors suggest that more studies on radiation carcinogenesis pathways are required.^[21]^

Part of the gross appearances of group A rectal cancer differed from that of other rectal cancers that were observed in this study. Patients in group A presented more ulcerative or infiltrative tumors (92.0% vs 77.8%) but fewer polypoid type tumors (8.0% vs 21.4%). There were 2 patients who presented with atypical deep ulcers, which may indicate that carcinogenesis pathways differ between radiotherapy-related and other rectal cancers. The gross picture of radiotherapy-related rectal adenocarcinoma is different from that generally observed in rectal cancer, and some patients present only rectal ulcerations that are not grossly tumor-like. Therefore, rectal biopsies of suspected lesions are suggested for identifying this subclinical malignancy. This observation was compatible with one previous Japanese study, Tamai report four radiation associated rectal cancer, 3 of 4 patients (75%) presented with diffuse infiltrative lesion without marked macroscopic tumor appearance.^[22]^

One significant finding of this study was the very low lymph node rate in secondary rectal cancer, and only 2 patients (8%) presented with regional lymph node metastasis. The possible reason for this observation could relate to lymphatic duct occlusion due to radiation fibrosis. A large number of patients developed low leg lymph edemas after pelvic radiotherapy due to pelvic lymph vessel occlusion.^[23]^ Occlusion of lymphatic vessels also block the spread of cancer cells. Another possible reason could be that radiotherapy-related tumors behave differently from non-radiation rectal cancer. In this study, we observed patients in group A had a higher portion (27.6%) of poorly or undifferentiated subtypes. The same observation was also found in post radiation osteogenic sarcoma of bone and soft tissues.^[24]^ Despite the low percentage of stage III rectal cancer, the OS of the patients in group A was still worse than that of the patients in the other groups. We suppose that the reason for this was that most patients in group A were older than group B. These patients also had a higher portion of stage IV disease, and more of them had died from causes other than rectal cancer. Despite the worse survival rates in the patients of group A, the average interval for secondary rectal cancer occurrence was 18 years; thus, the benefit from radiation therapy for cervical cancer control should not be underestimated.

There are 2 limitations to this study. First, the true incidence of radiation-associated secondary rectal cancer is hard to differ from that of sporadic secondary rectal cancer. Genetic mutations and other known risk factors (such as smoking and low fiber intake) have been associated with an increased incidence of second primary cancers. Previous studies have defined the clinical criteria of radiation-associated secondary rectal cancer, including a tumor in the radiation area, chronic inflammation near the tumor, and a long interval between the radiotherapy and cancer occurrence. However, it is still difficult to conclude that the secondary rectal cancers that meet those criteria were directly induced by the radiation. Second, the duration between the pelvic radiation and rectal cancer occurrence was very long; hence, the details of the radiation protocol and dose were mission in many patients. We are unable to assess the relationship between the radiation protocol and secondary rectal cancer characteristics. The radiotherapy protocol for cervical cancer in modern practice had been changed to focus more on the cervix and to allow less dosage to the rectum or other pelvic organs, as well as an accurate estimation of the radiation dose. Thus, it is possible that the incidence of secondary rectal cancers will be decreased using the present radiation technique.

In conclusion, the patients with rectal cancer developed after radiotherapy for cervical cancer usually presented with abnormal abdominal symptoms, such as proctitis, cystitis, or rectal fistula. The clinicopathologic characteristics of these patients were different from those of patients not treated with radiation therapy for cervical cancer, such as poor survival, older age, higher rates of anemia, gross ulcerative lesions, higher colostomy rates due to pelvic fibrosis, and a lower rate of regional lymph node metastases.

## Author contributions

**Conceptualization:** Hsinyuan Hung, Jengfu You.

**Data curation:** Hsinyuan Hung, Jengfu You, Paoshiu Hsieh, Sumfu Chiang, Chengchou Lai, Wensy Tasi, Chienyuh Yeh, Yihjong Chern, Yujen Hsu, Jy-Ming Chiang.

**Supervision:** Jengfu You, Jyming Chiang.

**Writing – orinial draft:** Hsinyuan Hung

**Writing – review & editing:** Hsinyuan Hung, Jengfu You.
